# Effects of fixed and removable space maintainers on halitosis

**DOI:** 10.1186/s12903-016-0297-6

**Published:** 2016-09-22

**Authors:** Elif Yıldızer Keriş, Didem Atabek, Kahraman Güngör

**Affiliations:** 1 Department of Radiology, Çanakkale Dentistry Hospital, 1700, Kepez, Çanakkale, Turkey; 2 Department of Pediatric Dentistry, Gazi University, Emek, Ankara, 06510 Turkey; 3 Department of Oral Diagnosis and Radiology, Gazi University, Emek, Ankara, 06510 Turkey

**Keywords:** Space maintainers, Halitosis, Oral hygiene

## Abstract

**Background:**

The current study evaluated the effects of fixed and removable space-maintainers on halitosis and oral health of children.

**Methods:**

Forty-three patients randomly selected between the ages 4–10 whom fixed or removable space maintainers were indicated in Gazi University, Faculty of Dentistry, Department of Pedodontics. The inclusing criteria were: caries-free (with/without restorations), with absence of periodontal diseases, without systematic disease, no mouth breathing and no use of antibiotics the 2-months period before the study. The patients were subdivided into two groups: the group treated with fixed space maintainers (group 1, *n* = 27), the group treated with removable space maintainers (group 2, *n* = 16). The the plaque index (PI), gingival index (GI), periodontal screening index (PSI), tongue coating index (TCI) records and the halitosis measurements were obtained at three time periods (T1: immediately after application, T2: 1 week after application, T3: 5 weeks after application). The measurement values of T1 served as control.

**Results:**

No statistically significant difference was found in the PI, GI, PSI, TCI and halitosis measurements between T1–T2, T2–T3 and T1–T3 in both appliance groups and between the groups (*p* > 0.05).

**Conclusions:**

The fixed and removable space maintainers did not affect oral health status and halitosis significantly.

## Background

Halitosis or oral malodor is an unlikeable or bad odor arising from the oral cavity, which is a common problem that effects social relationships [1].

The etiological factors of halitosis include extrinsic and intrinsic causes [2, 3]. The extrinsic causes are using tobacco, alcohol and some foods [4–6]. The intrinsic causes contain intra-oral and extra-oral causes [7]. Intra-oral causes are related with oral hygiene problems and oral diseases such as tongue coating, periodontal disease, extensive dental caries, pericoronitis, impacted food, unclean denture, stomatitis, xerostomia and habitual mouth breathing [1, 8–10]. Extra-oral causes are systemic diseases and some medications that effects the oral odor [1, 8, 11]. Oral conditions are responsible for halitosis in nearly 90 % of all cases [2, 12].

The three main methods for measuring and assessing the halitosis are organoleptic measurement, gas chromatography, and sulfide monitoring. In organoleptic method, the physician is smelling the exhaled air of mouth and nose while the patient is breathing and speaking and halitosis is assessed using a score of 0–5. Organoleptic assessment is considered as a suitable method for detecting halitosis but has some disadvantages such as being subjective [13]. Gas chromatography and portable sulfide monitor have been developed to evaluate halitosis objectively. Methyl mercaptan, hydrogen sulfide, butyric acid, proprionic acid and valeric acid are called as volatile sulphur components (VSCs) and these components are major cause of halitosis [14]. These components are occured as a result of the anaerobic bacteria by metabolizing different cells/tissues located in the mouth [14, 15]. Portable sulphur monitors (Halimeters) and gas chromatography measure the total concentration of sulphur compounds.

Space main-tainers are found to be associated with increased plaque accumulation [16]. Furthermore, the correlation between plaque accumulation and halitosis is clearly demonstrated in the literature [17]. Numerous studies have reported on the increased halitosis in patients using orthodontic appliances [18, 19]; however, there are no published studies relating to halitosis in conjunction with the use of fixed and removable space maintainers. Thus, the present study was aimed at investigating the effects of fixed and removable space maintainers on periodontal health and on halitosis.

## Methods

Ethical approval for this study was obtained from the Ethical Committee of the Faculty of Dentistry, University of Ankara. The study population was composed of 43 patients randomly selected between the ages 4–10 whom fixed or removable space maintainers were indicated in Gazi University, Faculty of Dentistry, Department of Pedodontics. All patients/parents were informed and their consent was given prior to entering the study. The inclusing criteria for this study were: caries-free (with/without restorations), with absence of periodontal diseases, without systematic disease, no mouth breathing and no use of antibiotics the 2-months period before the study.

The patients were subdivided into two groups randomly: the group treated with fixed space maintainers (group 1, *n* = 27, 14 girls, 13 boys), the group treated with removable space maintainers (group 2, *n* = 16, nine girls, seven boys). Oral hygiene instructions were given to all patients and their parents 1 week before the beginning of the study by a pediatric dentist (D.A.) and patients were asked to brush their teeth and tongue supervised by their parents. To standardize the patient population, only band-and-loop type space maintainers were included in the fixed space maintainer group. All removable space maintainers were made of an acrylic base and retention elements (vestibul arch, Adam’s and C clasps).

At three time periods (T1: immediately after application, T2: 1 week after application, T3: 5 weeks after application), patients were clinically examined by the same pediatric dentist (D.A.) trained in the use of the assessment clinical parameters used in the study and the halitosis measurements were obtained. The measurement values of T1 served as control.

### Clinical evaluation

Immediately prior to placement of the space maintainers, the plaque index (PI), gingival index (GI) [20], periodontal screening index (PSI) [21] (using a manual North Carolina 15 periodontal probe) were measured at the mid facial, mid lingual, and buccal line angles of the teeth which are one each of the maxillary right or left molars and and the antagonist teeth, mandibular right or left molars and the antagonist teeth, maxillary or mandibular incisors and the antagonist teeth.

Tongue coating was recorded according to the Miyazaki tongue coating index (TCI) [14].

### Halitosis measurements

For determininig halitosis and the level of detection, measurements were done according to organoleptic assessment and using portable sulphur monitor (Halimeters, Interscan corporation, Chatsworth, CA, USA).

The subjects were instructed to refrain from eating (especially garlic and onion), drinking coffee, eating mints, using minted chewing gum or scented oral hygiene products, and rinsing their mouths for 2 h before the examination. All measurements were recorded between 8:30 and 11:30 h (before lunch).

VSC concentrations were measured using a Halimeter (Model No. RH17R; Chatsworth, CA). The subject was asked to close his or her mouth and to breathe through the nose for 3 min before the Halimeter reading was taken. It was used according to the manufacturer’s instructions with a newly calibrated detector. The subject was asked not to exhale or inhale while the Halimeter reading was collected. The highest score was recorded, and the procedure was repeated twice at 3-min intervals, resulting in three Halimeter readings, from which a mean odor score was calculated. The mean value was calculated in parts per billion (ppb) for each patient. According to the manufacturer, halitosis is present at a VSC value >110 ppb.

### Statistical analysis

Power analysis showed that for a power of 0.80 with an α error of 0.05, 16 patients would be required for each group.

The records were statistically analyzed by using SPSS (version 17.0; SPSS Inc, Chicago, Ill). The Kolmogorov-Smirnov test was applied to test for normal distribution. Comparisons of parameters in the groups and times were evaluated according to two-way variance analysis (ANOVA), Chi-Square test and Fisher’s Exact test and and *p* < 0.05 set for level of significance.

## Results

### Clinical measurements

**Fig. 1:**
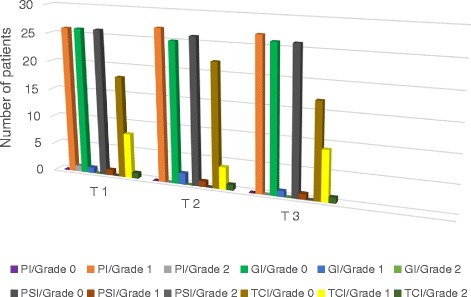
Values for clinical parameters at T1, T2 and T3 of the fixed applience group

**Fig. 2:**
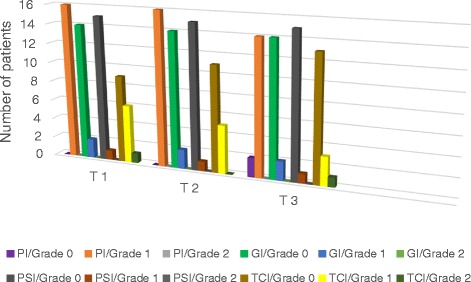
Values for clinical parameters at T1, T2 and T3 of the removable applience group

Values for clinical parameters at T1,T2 and T3 of the groups are provided in Table 1 and Figs. 1 and 2.

**Table 1:** Values for clinical parameters at T1, T2 and T3 of the groups

	T1	T2	T3
Fixed applience group			
PI (Plaque Index)			
Grade 0: No plaque	0	0	0
Grade 1: Not visible thin coating of plaque which is only visible after using the probe	26	27	27
Grade 2: Moderate accumulation of plaque, visible with the naked eye, but not filling interdental space	1	0	0
Grade 3: Abundance of plaque, filling interdental space	0	0	0
GI (Gingival Index)			
Grade 0: No inflammation.	26	25	26
Grade 1: Mild inflammation, slight change in color, slight edema, no bleeding on probing.	1	2	1
Grade 2: Moderate inflammation, moderate glazing, redness, bleeding on probing.	0	0	0
Grade 3: Marked redness and edema, ulceration with tendency to spontaneous bleeding.	0	0	0
PSI (Periodontal Screening Index)			
Grade 0: No bleeding on probing, no pathologic pocket, no calculus	26	26	26
Grade 1: Bleeding on probing up to 1 mm	1	1	1
Grade 2: Calculus and no pathologic pocket	0	0	0
Grade 3: Probing depth 3.5–5.5 mm	0	0	0
Grade 4: Probing depth > 5.5 mm	0	0	0
TCI (Tongue Coating Index)			
Grade 0: No visible coating	18	22	17
Grade 1: Less than a third of tongue dorsum is covered	8	4	9
Grade 2: Less than two thirds of tongue dorsum is covered	1	1	1
Grade 3: More than two thirds of tongue dorsum is covered	0	0	0
Removable applience group			
PI (Plaque Index)			
Grade 0: No plaque	0	0	2
Grade 1: Not visible thin coating of plaque which is only visible after using the probe	16	16	14
Grade 2: Moderate accumulation of plaque, visible with the naked eye, but not filling interdental space	0	0	0
Grade 3: Abundance of plaque, filling interdental space	0	0	0
GI (Gingival Index)			
Grade 0: No inflammation.	14	14	14
Grade 1: Mild inflammation, slight change in color, slight edema, no bleeding on probing.	2	2	2
Grade 2: Moderate inflammation, moderate glazing, redness, bleeding on probing.	0	0	0
Grade 3: Marked redness and edema, ulceration with tendency to spontaneous bleeding.	0	0	0
PSI (Periodontal Screening Index)			
Grade 0: No bleeding on probing, no pathologic pocket, no calculus	15	15	15
Grade 1: Bleeding on probing up to 1 mm	1	1	1
Grade 2: Calculus and no pathologic pocket	0	0	0
Grade 3: Probing depth 3.5–5.5 mm	0	0	0
Grade 4: Probing depth > 5.5 mm	0	0	0
TCI (Tongue Coating Index)			
Grade 0: No visible coating	9	11	13
Grade 1: Less than a third of tongue dorsum is covered	6	5	3
Grade 2: Less than two thirds of tongue dorsum is covered	1	0	0
Grade 3: More than two thirds of tongue dorsum is covered	0	0	0

No statistically significant difference was found in the PI, GI, PSI and TCI variable between T1–T2, T2–T3 and T1–T3 in both appliance groups and between the groups (Chi-Square, *p* = 1.00 > 0.05). Grade 1 of the plaque index was found with 26 of 27 patients and one of the patients recorded as grade 2 in fixed space maintainer group, and all af the patients with removable space maintainer recorded as grade 1 at T1. However the plaque scores decreased over time.

PSI, GI scores were found mostly grade 0 and were found under grade 2 with almost all of the patients of both appliance groups over all time.

TCI was decreased by time in removable space maintainer group. Only one patient’s TCI scores with fixed sapace maintainer increased at T3.

### Halitosis measurements

**Fig. 3:**
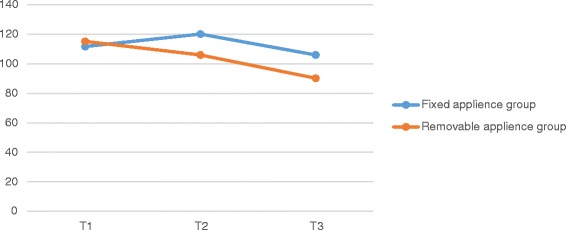
Mean halitosis scores of groups at three evaluation times

No statistically significant difference was found in halitosis values between T1–T2, T2–T3 and T1–T3 in both appliance groups (Two-Way ANOVA, *p* = 0.917 > 0.05) and between groups (Two-Way ANOVA, *p* = 0.709 > 0.05). In both groups, halitosis values decreased at T3 but statistically non significant (Table 2 and Fig. 3).

**Table 2:** Mean halitosis scores of groups at three evaluation times

	T1 Mean ± SD	T2 Mean ± SD	T3 Mean ± SD
Fixed applience group	111,7037 ± 63,6884	120,1852 ± 73,9491	105 ± 46,7596
Removable applience group	115,125 ± 89,0654	105,9375 ± 53,7407	90,25 ± 50,0473

## Discussion

The use of space maintainers has been stated to result in an increased plaque accumulation, which can lead to periodontal disease [16, 22]. Previous studies have revealed that the presence of gingivitis or periodontitis increases the risk of developing halitosis [23–25]. There are limited numbers of studies evaluating the effects of space maintainers on periodontal status [16, 26] however, of these, none of them investigated the effects of space maintainers on halitosis.

In this study patients were instructed to brush both teeth and tongue because previous studies indicated that increase in tongue coating and periodontal diseases were two major causes for halitosis [27, 28].

In previous studies, the effects of space maintainers and orthodontic appliances on periodontal indices were evaluated with longer periods of follow-up [16, 26, 29]. Altough, following-up with longer periods is required to evaluate more accurately, patient cooperation, motivation for oral hygiene procedures, and eating habits can change with time [30]. Therefore, based on previous studies, this study was terminated in the 5th week [30–33].

According to our results for plaque index, grade 1 of the plaque index was found with 26 of 27 patients and one of the patients recorded as grade 2 in fixed space maintainer group, and all af the patients with removable space maintainer recorded as grade 1 at T1. It seems it was difficult to perform adequate oral hygiene despite careful instruction because younger patients can remove plaque over the surface much worse. However the plaque scores decreased over time. It could be due to the Hawthorne effect, a phenomenon where subjects improve or modify their behavior when they know they are being observed [34] .

Compatible with decreasing in plaque scores, the results of this study showed no significant differences in PSI, GI values during the treatment/observation time in all the groups. Anyway, our patients’ PSI, GI scores were under grade 1 mostly over all time.

Studies which are evaluating the effects of space maintainers on periodontal conditions are very rare [16, 26]. Methodologies of these previous studies are different from our study because these studies compared the plaque, gingival and bleeding on probing index scores in regions where space maintainers were present (test teeth) with in regions where space maintainers were not present (opposing teeth). We did not diveded the teeth as test and opposing during statistical analysis because halitosis measurements would be affected by periodontal status both of the teeth which were related and unrelated to space maintainers. One of these previous studies by Arikan V et al. [26] revealed that the removable space maintainers did not affect plaque accumulation in test teeth. In addition, they found that the plaque scores of fixed space maintainers in test teeth were lower at time of 6th month than baseline. At the same time, the authors reported that the gingival indices and bleeding on probing scores did not significantly differ between the regions with and without removable space maintainers, and there was not any negative effect of fixed space maintainers on periodontal status in test teeth at 6th month. Although the statistical analysis vary from those in our study, their results are in accordence with our results. In contrast to our study, the other previous study by Arikan F et al. [16], observed that both fixed and removable space maintainers cause an increase in plaque accumulation in all teeth, and an increase in bleeding index and pocket depth in test teeth, nevertheless removable appliance group showed less increase. Overall decreases in the index scores in our study may result from good oral hygiene unrelated to space maintainer use.

The effects of removable and fixed orthodontic appliances on periodontal health have been studied previously. The results of this present study are incompatible with the previous studies that suggest a strong relationship between orthodontic appliance treatment and plaque accumulation and increasing periodontal indices [18, 30, 33]. Similiarly with our findings, other studies reported decreasing in plaque indices and improvement of periodontal health [29, 35]. These different results may be related to the study design such as the different age groups, appliances used, observation time, the level of oral hygiene of the subjects and statistical analyses.

Tongue cleaning was in accordance with decreased halitosis scores in this study. Recent studies showed that tongue cleaning may positively affect the halitosis scores [29].

The organoleptic assessment is preferable for the daily practice for diagnosis of bad breath because it is simple to perform and does not require a device. But organoleptic method has some advantages such as not being reproducible, having crossinfection risks and can be affected by the examiner [36–38]. It has been reported that gas chromatography is reliable, objective and reproducible method for detecting halitosis [39]. However, these devices are complex, not portable and expensive, requires the user’s experience [40]. In order to overcome these practical drawbacks, portable sulphur monitors (Halimeters) is preferred for routine use in the dental clinic. The Halimeter does not need experienced personnel and easy to perform [39]. But Halimeter can only measure the VSCs, the organoleptic method is superior for assessing halitosis caused by non-sulphide components [13]. However, recent studies have concluded that the recorded data of Halimeter is correlated with the data of organoleptic method for diagnosing halitosis [41].

In both groups, halitosis values decreased at T3 but statistically non significant. Decreased halitosis scores were in accordance with improved oral healh status. It was not possible to compare the results of our study with other experiments, since the documents about the effect of space maintainers on the halitosis of children is lacking and studies are mostly performed on children receiving orthodontic treatment. It is demonstrated that orthodontic treatment effects halitosis. Kaygısız et al. [29] evaluated the effect of fixed orthodontic treatment on oral malodor. They evaluated halitosis values of patients treated with fixed orthodontic appliances. The authors found nonsignificant decreases 4 and 8 weeks after bonding in the fixed orthodontic appliance group, which is similar with our study.

A limitation of this study could be the lack of data on the organoleptic scores of the patients. Assessing halitosis by organoleptic method appears sensible, it has been suggested that halitosis should be diagnosed with two different methods, organoleptic and instrumental measurements [41]. Because of the cross-infection risks of organoleptic measurement, only Halimeter used in this research.

## Conclusions

The fixed and removable space maintainers did not affect oral health status and halitosis significantly.

## Abbrevıatıons

ANOVA: Two-way variance analysis
GI: Gingival index
PI: Plaque index
ppb: parts per billion
PSI: Periodontal screening index
T1: Immediately after application
T2: 1 week after application
T3: 5 weeks after application
TCI: Tongue coating index
VSCs: Volatile sulphur components
